# Activation of the WNT-BMP-FGF Regulatory Network Induces the Onset of Cell Death in Anterior Mesodermal Cells to Establish the ANZ

**DOI:** 10.3389/fcell.2021.703836

**Published:** 2021-11-08

**Authors:** Martha Elena Díaz-Hernández, Claudio Iván Galván-Hernández, Jessica Cristina Marín-Llera, Karen Camargo-Sosa, Marcia Bustamante, Sabina Wischin, Jesús Chimal-Monroy

**Affiliations:** Instituto de Investigaciones Biomédicas, Universidad Nacional Autónoma de México, Ciudad Universitaria, México, Mexico

**Keywords:** limb development, ANZ, Fgf signaling, Wnt signaing, BMP signaling, programmed cell death, morphogenesis, Anterior Necrotic Zone

## Abstract

The spatiotemporal control of programmed cell death (PCD) plays a significant role in sculpting the limb. In the early avian limb bud, the anterior necrotic zone (ANZ) and the posterior necrotic zone are two cell death regions associated with digit number reduction. In this study, we evaluated the first events triggered by the FGF, BMP, and WNT signaling interactions to initiate cell death in the anterior margin of the limb to establish the ANZ. This study demonstrates that in a period of two to 8 h after the inhibition of WNT or FGF signaling or the activation of BMP signaling, cell death was induced in the anterior margin of the limb concomitantly with the regulation of *Dkk*, *Fgf8,* and *Bmp4* expression. Comparing the gene expression profile between the ANZ and the undifferentiated zone at 22HH and 25HH and between the ANZ of 22HH and 25HH stages correlates with functional programs controlled by the regulatory network FGF, BMP, and WNT signaling in the anterior margin of the limb. This work provides novel insights to recognize a negative feedback loop between FGF8, BMP4, and DKK to control the onset of cell death in the anterior margin of the limb to the establishment of the ANZ.

## Introduction

Programmed cell death (PCD) is essential to regulate the final morphology and sculpting limbs (Pajni-Underwood et al., 2007; Montero et al., 2020). PCD participates in separating digits and zeugopodial elements (Hinchliffe and Thorogood, 1974; Zuzarte-Luis and Hurle, 2005). The anterior and posterior margins of the avian limb are associated with digit reduction (Saunders and Gasseling, 1962; Zuzarte-Luis and Hurle, 2002; 2005). Those regions are called the anterior necrotic zone (ANZ) and posterior necrotic zone (PNZ) (Saunders and Gasseling, 1962). Meanwhile, PCD in the interdigital regions takes part in species with free digits.

The process of cell death is under the control of the apical ectodermal ridge (AER); it is well known that limb truncation and massive cell death occur after the elimination of the AER, demonstrating that cell survival of mesodermal cells depends on the signals from this epithelium. The molecular analysis of AER indicates that Fibroblast Growth Factor (FGF) family members expressed in the AER govern cell proliferation and cell survival. FGF8 signaling protects the mesodermal cells from cell death (Niswander et al., 1993; Sun et al., 2002; Mariani et al., 2008; Ten Berge et al., 2008; Mariani et al., 2017). Mutant mice for the *Fgf8* gene or the blockade of the function of FGF signaling induces cell death in the mesodermal cells (Montero et al., 2001; Sun et al., 2002; Mariani et al., 2008; Mariani et al., 2017). FGF8 participates promoting a positive feedback loop, inducing the expression of *Fgf10* in the mesoderm whose interaction with the FGF receptor 2b (FGFR2b) in the AER promotes the expression of *Wnt3a* (Xu et al., 1998; Danopoulos et al., 2013; Haro et al., 2014; Jin et al., 2018). WNT3A induces the expression of transcription factors *Sp6* and *Sp8* that induce *Fgf8* expression in the AER (Kawakami et al., 2001; Kawakami et al., 2004). WNT signaling from the AER participates in maintaining the undifferentiated state of mesodermal cells under AER (Ten Berge et al., 2008). The function of WNT-ß catenin signaling may be blocked by DKK, an antagonist of this signaling pathway. Gene deletion of DKK results in cell death inhibition, and *Dkk* expression is observed in cell death regions during limb development (Grotewold et al., 1999; Mukhopadhyay et al., 2001; Grotewold and Ruther, 2002a; b).

During limb development, the bone morphogenetic proteins (BMP) signaling is another pathway controlling cell death. *Bmp2, Bmp4,* and *Bmp7* expression are observed in the interdigital regions and the anterior and posterior margins of the limb (Hogan, 1996; Yokouchi et al., 1996; Macias et al., 1997; Chen and Zhao, 1998; Merino et al., 1999; Salas-Vidal et al., 2001; Zuzarte-Luis et al., 2004). The implantation of BMP-soaked beads promotes cell death in the interdigital tissue, whereas the blockade of BMP signaling with NOGGIN or GREMLIN inhibits it (Ganan et al., 1996; Macias et al., 1997; Zuzarte-Luis et al., 2004). BMP stimulates the SMAD1/5/8 signaling pathway in blood vessels and mesodermal cells, promoting cell death in mesodermal cells, and inhibiting *Fgf8* expression in the AER (Zuzarte-Luis et al., 2004; Monteiro et al., 2008; Abarca-Buis et al., 2011).

High levels of FGF signaling in the interdigital mesoderm downregulate *Bmp* genes, inhibiting cell death (Montero et al., 2001; Hernandez-Martinez et al., 2009). However, FGF2 signaling promoted cell death induced by BMP proteins, suggesting that FGF also works in a feedback loop with BMP signaling (Montero et al., 2001). Mesodermal cells become competent to signaling pathways that control cell fate when WNT and FGF signals are depleted underneath AER (Ten Berge et al., 2008). If cells receive cell death-promoting factors, presumably BMPs, cells enter the cell death program (Montero et al., 2001; Hernandez-Martinez et al., 2009). Otherwise, the cell differentiation program begins if mesodermal cells receive chondrogenic signals (Chimal-Monroy et al., 2003; Montero et al., 2008; Marin-Llera et al., 2019). Thus, FGF and WNT signaling together with BMP signaling establish a well-known regulatory network to control the undifferentiated state, cell proliferation, and cell survival during limb development (Niswander et al., 1993; Montero et al., 2001; Sun et al., 2002; Mariani et al., 2008; Ten Berge et al., 2008; Hernandez-Martinez et al., 2009; Mariani et al., 2017). The participation of this regulatory network is better known during the PCD in the interdigital regions than in the ANZ or PNZ. A previous study demonstrated that a BMP-pulse of 4 h was sufficient to induce cell death in the anterior margin of the limb (Abarca-Buis et al., 2011). Notably, TUNEL-positive cells show no co-localization of nuclear phosphorylated SMAD1/5/8 proteins suggesting that BMP signaling participates in a molecular cascade in the ANZ, culminating in cell death (Abarca-Buis et al., 2011).

Because a short pulse of BMP is sufficient to induce cell death in the anterior margin of the limb, this work aimed to determine how the regulatory network integrated by FGF, BMP, and WNT signaling pathways participate in the establishment of the ANZ. The results presented here demonstrated that inhibition of WNT or FGF signaling or the activation of BMP signaling during a short period is sufficient to induce cell death in the anterior margin of the limb and to regulate *Dkk*, *Fgf8,* and *Bmp4* expression. Thus, the regulatory network of the FGF-BMP-WNT signaling pathway induces cell death in the anterior margin of the limb to establishing the ANZ.

## Material and Methods

### Ethics

This protocol was reviewed and approved by the Institutional Review Board for the Care and Use of Laboratory Animals of the Instituto de Investigaciones Biomédicas, Universidad Nacional Autónoma de México (UNAM, Mexico City, Mexico).

### Eggs and Embryo Manipulations

Fertilized White Leghorn chicken eggs (ALPES, Puebla, Mexico) were incubated at 38°C and staged according to Hamburger and Hamilton (1951). Eggs were windowed to expose the right limb at developing stages from 22 to 25 HH for experimental procedures. Heparin beads (Cat. H6508, Sigma-Aldrich, St. Louis, MO, United States) or in Affigel (Bio-Rad Laboratories, Hercules, CA) were soaked in 1 mg/ml FGF8 (cat. 100-25A), FGF10 (100-26), 1 mg/ml DKK (cat. 120-30) BMP4 (cat. 120-05), BMP7 (cat. 120-30P) or 2 mg/ml NOGGIN (cat. 120-10C) from Peprotech, Mexico City, Mexico. Ag1-X2 ionic exchange beads (Cat. 1401231, Sigma-Aldrich, Mexico) were soaked in 4 mg/ml SU502 and placed in the ANZ of embryonic limbs. Manipulated embryos were incubated for short times according to the experiments and processed for lysotracker staining, *in situ* hybridization, or both.

### RNA Probes and in Situ Hybridization

RNA antisense probes were labeled with UTP-digoxigenin (11209256910, Roche Applied Science, Indianapolis, IN, United States) and used for whole-mount *in situ* hybridization (ISH) as described previously (Merino et al., 1998). Samples were treated with 60 μg/ml proteinase K for 25 min at 21°C for *Bmp7*, *Fgfr1*, *Fgfr2*, *Fgfr3*, *Mkp3*, *Msx2*, and *Wif*. *Bambi* required 70 μg/ml proteinase K for 28 min at 25°C; 60 μg/ml was used for 22 min at 21°C for *Bmp4* and *Dkk*. *Fgf8* was treated with 15 μg/ml for 20 min at 21°C. The hybridization temperature was 68°C, and post-hybridization washes were at 70°C for all genes. The signal of ISH was visualized with BM-Purple substrate for alkaline phosphatase (Roche Applied Science). Images were acquired with the Nikon Stereoscope Fluorescence Microscope SMZ1500 (Nikon Corporation, NY, United States) or in AxioZoom V.16 microscope (Carl Zeiss, Oberkochen, Germany) using Zen lite software (Carl Zeiss, Oberkochen, Germany).

### Detection of Cell Death With Lysotracker and Neutral Red Staining

Embryonic limbs were incubated in 1 μM LysoTracker Red DND-99 (Cat. L7528, Molecular Probes) at 37°C for 15 min. Samples were rinsed twice in PBS and fixed in 4% PFA overnight at 4°C. Some samples were also processed for ISH as described previously (Abarca-Buis et al., 2011). After ISH, limb buds were dehydrated in increasing methanol-PBS-Tween series and cleared with 2:1 benzylic alcohol: benzyl benzoate solution for 1 h each (Parish, 1999). For Neutral Red staining, limbs were isolated, washed in PBS, and stained with 2% Neutral Red in PBS time at 37°C. Images were acquired with a Nikon Stereoscope Fluorescence Microscope SMZ1500 (Nikon Corporation, NY, United States) and Fluorescence Microscope Axio Zoom. V16 Carl Zeiss, Göttingen, Germany.

### Real-Time RT-PCR

RNA extractions were performed with NucleoSpin RNA (Macherey-Nagel, cat. no. 740955, Düren, Germany), and retrotranscription of total RNA was achieved using the RevertAid RT kit (Thermo Fisher Scientific, cat. no. K1691, Waltham, MA, United States) according to the manufacturer’s instructions. Expression levels were analyzed using a real-time PCR system and quantified with SYBR green (Thermo Fisher). The *Rpl13* gene was used as a normalizer. The expression level was evaluated relative to a calibrator according to the 2^−(ΔΔCt)^ equation. Each value represented the mean ± SD of three independent experiments and was analyzed using Student’s *t*-test. Statistical significance was set at *p* < 0.05.

## Results

### Anterior Necrotic Zone Appears by the Progressive Loss of the Apical Ectodermal Ridge

**FIGURE 1:**
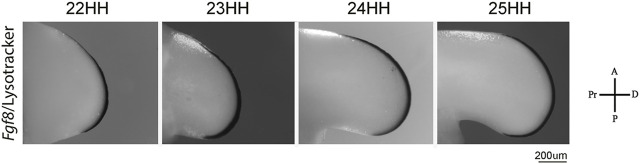
Progressive loss of the apical ectodermal coincides with the apparition of cell death in the anterior margin of the limb. *In situ* hybridization of *Fgf8* gene expression and cell death pattern evidenced by lysotracker stain at limb development stages from 22 to 25 HH. Notice that cell death occurs in regions in which *Fgf8* expression is disappearing.

To associate the gene expression pattern of *Fgf8*, a survival signal, and the presence of cell death in the ANZ during limb outgrowth, embryonic limbs from 22HH to 25HH were stained with lysotracker and hybridized for *Fgf8* (Figure 1). The results indicated that cell death was observed in the anterior margin of the limb from stage 23 HH to 25 HH (Figure 1). Concurrently as the limb outgrowth occurs, the *Fgf8* expression is gradually downregulated in the AER from the proximal to the distal region, near the ANZ. Thus, these results exhibited a boundary between the downregulation of *Fgf8* from the AER and the appearance of the ANZ while progressive cell death in mesodermal cells is observed (Figure 1).

### FGF-, BMP-, and WNT-Signaling Pathways Are Active in the Anterior Margin of the Limb Bud When the ANZ Is Established

**FIGURE 2:**
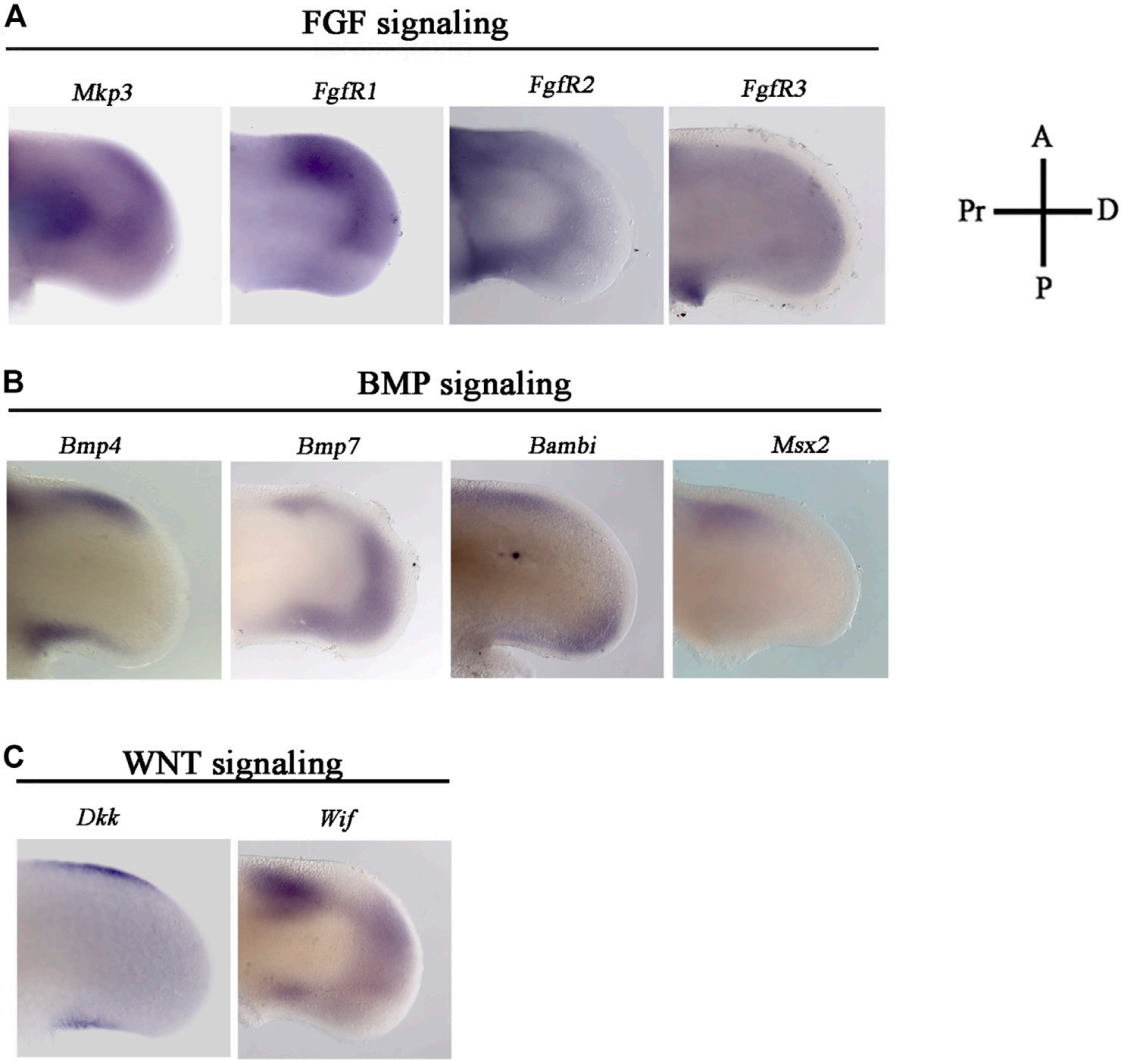
Gene expression pattern of effector genes related with WNT, FGF, BMP signaling in the ANZ and PNZ. Cell death pattern and *in situ* hybridizations of **(A)** *Mkp3, Fgfr1, Fgfr2, Fgfr3, Bmp4, Bmp7, Bambi, Msx2, Dkk, and Wif.* They were evaluated in the anterior margin of the limb at developing stage 24 HH. **(A)** *Mkp3, Fgfr1*, *Fgfr2*, and *Fgfr3* are genes related to FGF signaling. **(B)** *Bmp4*, *Bmp7*, *Bambi,* and *Msx2* related to BMP signaling, whereas **(C)** *Dkk, Wif,* related to WNT signaling. Notice the dynamic expression of all genes in the ANZ.

The association of the expression patterns of the genes related to FGF-, BMP-, and WNT-signaling pathways with the boundary observed between *Fgf8,* and the appearance of progressive cell death was studied at the 24 HH stage. At this developing stage, cell death and *Fgf8* expression are precisely located at neighboring positions (Figure 2), allowing us to study how FGF-, BMP-, and WNT-signaling pathways regulate the onset of cell death and the appearance of ANZ in the developing limb.

The genes related to FGF signaling, such as *Mkp3, a* target of this signaling pathway, and the three receptors of FGF, *Fgfr1*, *Fgfr2*, and *Fgfr3* (Figure 2A) were evaluated. *Mkp3* and *Fgfr1* were expressed in the distal part of the anterior margin and in the anterior half of the distal position. Besides, *Mkp3* was expressed in the central part of the proximal region. *Fgfr2* was mainly expressed in both anterior and posterior margins, whereas *Fgr3* was slightly expressed in an extended way in the mesoderm (Figure 2A).

Regarding BMP signaling, the expression of *Bmp4*, *Bmp7*, and *Bambi* was evaluated. *Bmp4* was expressed in both anterior and posterior margins of the limb (Figure 2B). In contrast, *Bmp7* expression was mainly localized in the most distal region of the limb riming undifferentiated region underneath AER and the anterior margin of the limb (Figure 2B). *Bambi* expression was observed preferentially in the proximal region of the anterior margin and the most distal region of the posterior margin of the limb (Figure 2B). In addition, as a marker of undifferentiated cells and regulated by BMP signaling, we evaluated the *Msx2* gene expression pattern. Results showed that it is expressed in the anterior margin of the limb (Figure 2B).

On the other hand, *Dkk* and *Wif* gene expression was analyzed as genes related to Wnt signaling (Figure 2C). *Dkk* gene expression was localized in the anterior and posterior margins of the limb (Figure 2). *Wif* gene expression was observed in all anterior and posterior margins and the distal region of the limb (Figure 2C). These results showed a dynamic gene expression pattern suggesting that all three signaling pathways are active during the process of cell death in the anterior and posterior limb margins.

### FGF, WNT, and BMP Signaling Control the Induction and Maintenance of Cell Death in the ANZ

**FIGURE 3:**
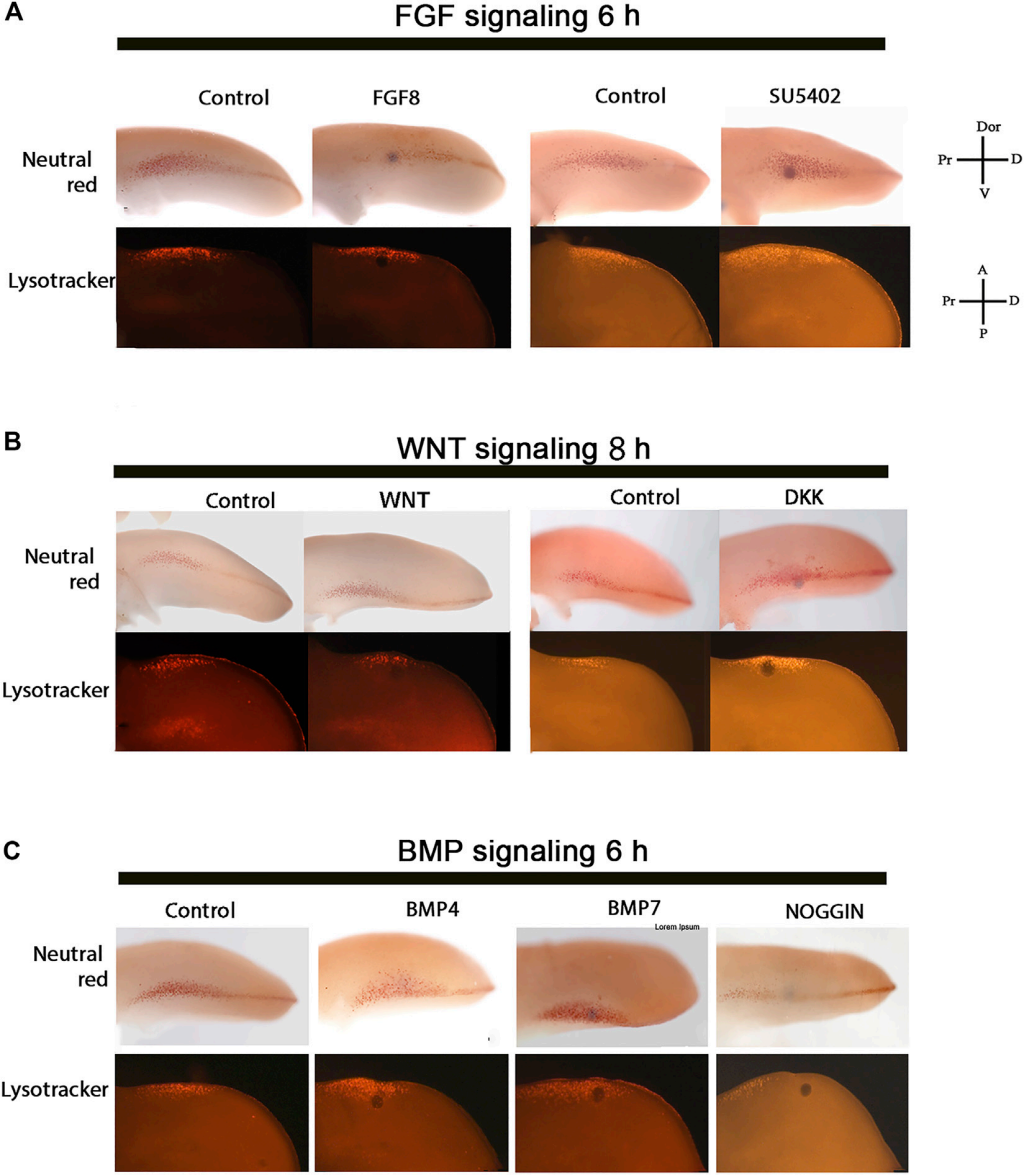
Control of PCD in the ANZ by FGF, WNT, and BMP signaling. The cell death pattern was evaluated in the ANZ at limb developing stage 24 HH after FGF, WNT, and BMP signaling treatments. **(A)** FGF8 treatment for 6 h did not modify the cell death pattern but inhibiting FGF signaling resulted in the promotion of PCD. **(B)** WNT3A treatment for 8 h did not alter the cell death pattern, but DKK-treatment to inhibit Wnt signaling induced cell death after 8 h. **(C)** BMP4- or BMP7- treatment for 6 h promoted cell death, whereas NOGGIN inhibited cell death after 6 h of treatment. Notice that the minimum time to trigger cell death in the ANZ is 6 h after inhibiting FGF or activating BMP signaling, whereas DKK needed 8 h to promote cell death. The images at the top of each line correspond to samples stained with Neutral Red, while the images at the bottom are stained with Lysotracker. The axis showed in A can be used for B and C.

Although the gene expression pattern is quite similar in the anterior and posterior margin of the limb, here was decided to determine the minimum time to promote cell death in the ANZ modulating the function of FGF, WNT, and BMP signaling pathways. As a first approach, FGF8-soaked beads or FGF signaling inhibitor (SU5402)-soaked beads were placed in the anterior margin of the limb at the 24 HH stage (Figure 3A). The results showed that FGF8 treatments did not inhibit cell death. In contrast, inhibiting FGF signaling for 6 h was sufficient to increase cell death in this region (Figure 3A). On the other hand, the minimal time to induce cell death after inhibiting WNT signaling with DKK-soaked beads in the anterior margin of the limb was 8 h. In contrast, as expected, WNT3A soaked beads did not inhibit cell death (Figure 3B).

It is known that a short pulse of BMP induces cell death in the ANZ (Abarca-Buis et al., 2011) and because the expression of the *Bmp4* and *Bmp7* is observed in the anterior margin. Here the cell death promoted by both proteins was evaluated. The results showed that cell death in the ANZ stimulated by BMP4 and BMP7 occurs after 6 h (Figure 3C) and is inhibited by NOGGIN after 6 h of treatment (Figure 3C). Taking together, these results suggest that the minimum time to trigger cell death in the ANZ is 6 h after inhibition of FGF and activation of BMP signaling or 8 h after inhibiting WNT signaling.

### FGF- and BMP-Signaling Are Coordinated to Regulate Cell Death in the ANZ

**FIGURE 4:**
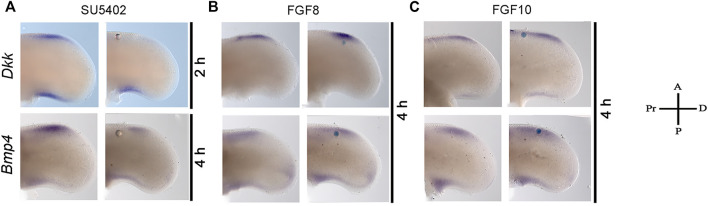
Inhibition of *Dkk* and *Bmp4* gene expression by treatment with an inhibitor on FGF signaling. Gene expression pattern of *Bmp4* and *Dkk* in the anterior margin of the limb at developing stage 24HH. **(A)** *Dkk* expression inhibition was observed after 2 h of treatment with SU5402, while Bmp4 was inhibited after 4 h. The gene expression pattern of *Dkk* and *Bmp4* was not modified at least 4 h after **(B)** FGF8- and **(C)** FGF10-treatment.

The inhibition of FGF or activation of BMP signaling triggers cell death after 6 h. Thus, to determine the relation between FGF and *Bmp4* and *Dkk* expression during the induction of cell death, the gene expression of *Bmp4* and *Dkk* was evaluated after inhibiting or activating FGF signaling (Figure 4). The results demonstrated that *Dkk* expression was inhibited after 2 h of treatment, whereas *Bmp4* inhibition occurred after 4 h (Figure 4A). However, after the administration of proteins FGF8 or FGF10 to activate FGF signaling, only FGF10 induced moderately *Bmp4* expression without important changes in the expression of *Dkk* and *Bmp4* by FGF8 (Figures 4 B, C). Thus, because the inhibition of FGF signaling for two or 4 h, downregulated *Dkk* and *Bmp4,* respectively, it is possible to suggest the expression of *Dkk* and *Bmp4* in the anterior margin of the limb depends on FGF signaling.

**FIGURE 5:**
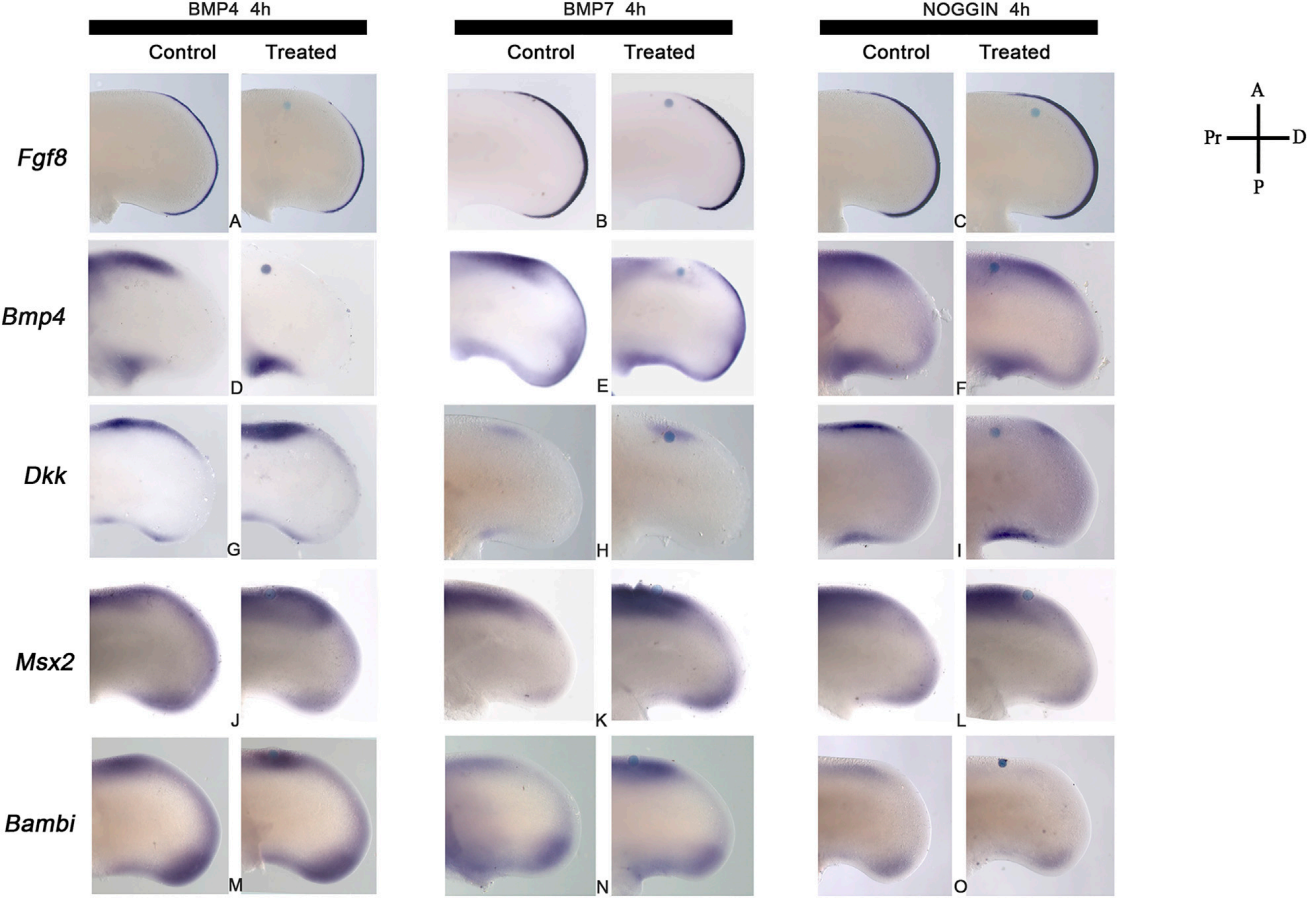
BMP signaling regulates *Fgf8, Bmp4, Dkk, Msx2, and Bambi* gene expression. Regulation of **(A–C)** *Fgf8*, **(D–F)** *Bmp4,* **(G–I)** *Dkk,* **(J–L)** *Msx2,* **(M–O)** *Bambi* gene expression in the anterior limb margin after BMP4, BMP7, or Noggin. *Fgf8* **(B, C)** and Bmp4 **(D, E)** are downregulated after 4 h of BMP4 or BMP7. Notice the stronger effect of BMP4 than BMP7. In contrast, NOGGIN-treatment maintained the expression of both genes after treatments. **(G–I)** Upregulation of Dkk gene expression after 4 h of BMP4- or BMP7-treatment and downregulation of *Dkk* after 4 h of NOGGIN treatment. **(J–L)** *Msx2* and **(M–O)** *Bambi* gene expression were upregulated by BMP4 and BMP7. In contrast, NOGGIN inhibited *Bambi* expression during the first 4 h of treatment but not *Msx2* [Panel **(L, O)**].

The next step was to determine the minimum time required for BMP signaling to regulate *Fgf8*, *Bmp4*, and *Dkk* gene expression in the anterior margin of the limb for promoting cell death (Figures 5A–I). The results showed that BMP4 or BMP7 proteins had regulated Fgf8, Bmp4, and Dkk gene expression differentially (Figures 5A,B,D,E,G,H). BMP4 protein inhibited *Fgf8* in the AER and *Bmp4* in the anterior margin of the limb after 4 h of treatment (Figures 5A,D). In contrast, BMP7 slightly affected *Fgf8* gene expression, and it faintly inhibited *Bmp4* gene expression (Figures 5B,E). Furthermore, NOGGIN-treatment effects on *Fgf8* expression were minor (Figure 5C), while *Bmp4* gene expression was not affected after 4 h (Figure 5F). Regarding *Dkk* gene expression, the treatment with BMP4 showed a more significant effect than BMP7 (Figures 5G,H). Blocking BMP signaling with NOGGIN demonstrated that *Dkk* gene expression depended on BMP signaling (Figure 5I). *Msx2* and *Bambi* are regulated by BMP signaling (Figures 5J–O). The results showed that *Msx2* was regulated by BMP or NOGGIN treatment (Figures 5J–L). In contrast, NOGGIN treatment inhibited *Bambi* expression entirely, but BMP4 or BMP7 induced it slightly at least 4 h (Figures 5M–O). These results showed that BMP signaling self-regulate *Bmp4* and regulates *Fgf8*, *Dkk*, *Msx2*, and *Bambi* gene expression at short times in the ANZ.

### Inhibition of FGF Signaling Is the Last Step in the Molecular Cascade of Cell Death

**FIGURE 6:**
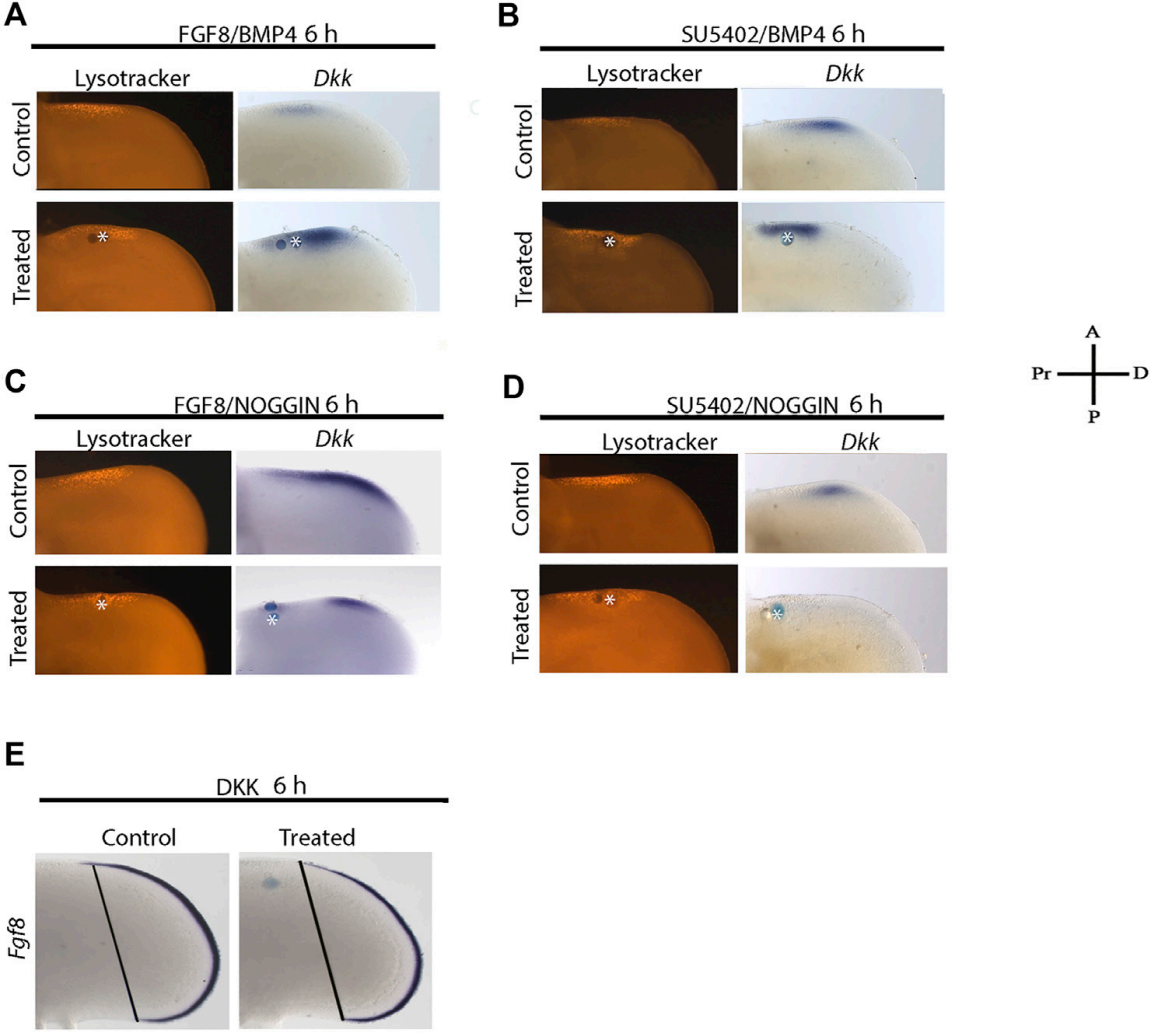
The last step to induce cell death is the inhibition of FGF signaling.Double treatments were done to promote or block FGF and BMP signaling for 6 h. Under these conditions, lysotracker staining and *Dkk* gene expression were evaluated. **(A)** Double FGF8 and BMP4 treatment promoted *Dkk* expression, and cell death was observed closer to the BMP bead. **(B)** Inhibiting FGF signaling in the presence of BMP4, *Dkk* was still expressed. **(C)** Treatment with NOGGIN and in the presence of FGF8 significantly inhibited *Dkk* expression, but it slightly diminished the area of cell death. **(D)** Double treatment inhibiting FGF and BMP signaling inhibited *Dkk* gene expression, but no cell death. **(E)** *Fgf8* gene expression is inhibited in response to DKK protein. Notice that the blue line of the right limb (DKK treatment)—representing the expression of Fgf8 from the limb posterior margin to the anterior limb margin—is shorter than the contralateral limb.

To determine the hierarchy of FGF and BMP signaling to promote cell death and regulate *Dkk* gene expression, we performed double treatments to promote or block FGF and BMP signaling for 6 h. Under these conditions, the dual treatment of FGF8 and BMP4 promoted *Dkk* expression inducing cell death closer to the BMP bead (Figure 6A). Nevertheless, if FGF signaling is inhibited in the presence of BMP4, *Dkk* is still expressed (Figure 6B). In contrast, if BMP signaling is blocked and FGF8 is present, the expression of Dkk was inhibited, and the area of cell death was slightly diminished (Figure 6C). The double blockade of FGF and BMP signaling inhibited *Dkk* gene expression. Under these conditions, cell death is still induced (Figure 6D). Finally, we evaluated the expression of *Fgf8* in the AER in the response of DKK protein that inhibited *Fgf8* (Figure 6E)*.* These results suggest that FGF signaling must be inhibited in the ANZ and is probably the last step in the molecular cascade to trigger PCD by BMP signaling.

### Dynamic of Gene Expression in Presumptive ANZ and ANZ

**FIGURE 7:**
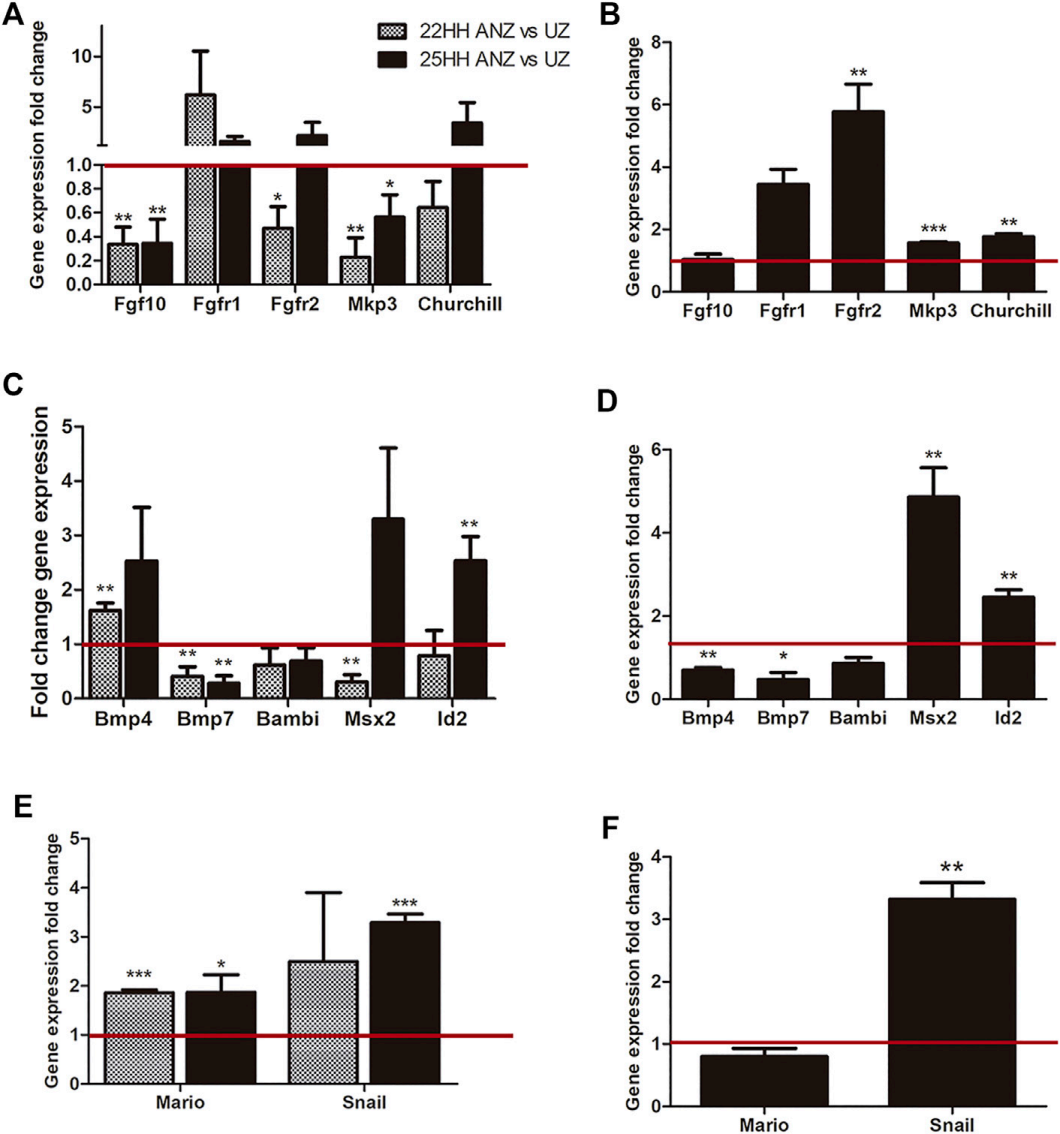
qRT-PCR analysis and comparison dynamics of gene expression in the presumptive ANZ and the ANZ. qRT-PCR of FGF-related genes (Fgf10, Fgfr1, Fgfr2, Mkp3, Churchill) and BMP-related genes (Bmp4, Bmp7, Bambi, Mario, Snail). **(A, C, E)** Comparison of gene expression between the presumptive ANZ in 22 HH vs its undifferentiated zone (UZ) and between the ANZ versus its UZ. **(B, D, F)** Analysis of gene expression in the ANZ at 25 HH stage relative to the presumptive ANZ at 22 HH stage (set to 1.0, red line). Data represent three independent experiments. Statistical significance was set as follows: ***p < 0.0001, **p < 0.005, *p < 0.05.

Once it was established that the onset of cell death requires the negative loop triggered by FGF signaling, a comparison of an expression profile of genes related to the maintenance of the undifferentiated stage and the commitment to cell death was performed (Figure 7). The tissue of limb primordia from 22 HH and the ANZ from 25 HH were dissected in two regions: 1) the mesodermal cells of the anterior margin at stage 22 HH (here called presumptive ANZ) or the ANZ at stage 25 HH, and 2) cells from the undifferentiated zone from both developing stages. It allowed us to clearly distinguish the location of genes expressed before establishing ANZ (stage 22 HH) and once established the ANZ (stage 25 HH). In both stages, the profile expression of this zone was compared with its respective undifferentiated zone.

Regarding genes related to the FGF signaling (Figures 7A,B), results showed that *Fgf10* expression was lower in the presumptive ANZ and ANZ than in the undifferentiated zone in both stages. *Fgfr1* tends to be upregulated in the presumptive ANZ than in the undifferentiated zone, but this study found no difference at both stages (Figure 7A). In the presumptive ANZ, *Fgfr2* expression was lower than in the undifferentiated zone, but at stage 25HH, the expression levels were similar in the ANZ and the undifferentiated zone (Figure 7A). However, comparing the levels of *fgf10*, *Fgfr1* and *Fgfr2* expressed between the presumptive ANZ at 22 HH and ANZ at 25 HH, the *Fgf10* expression did not show significant changes (Figure 7B). In contrast, the expression levels of both receptors increased at stage 25HH than a stage 22HH, although only *Fgfr2* increased significatively (Figure 7A). Next, the expression of Mkp3 and Churchill was evaluated, two target genes of FGF signaling (Kawakami et al., 2003; Sheng et al., 2003). In the presumptive ANZ and ANZ, the expression levels of *Mkp3* decrease compared to the undifferentiated zone, while *Churchill* has no significant changes (Figure 7A). Moreover, comparing the expression levels of both genes, they had a higher level in the ANZ than in the presumptive ANZ (Figure 7B). These data showed an interesting dynamic of FGF signaling.

The next group of genes analyzed was *Bmp4, Bmp7*, *Bambi*, and those regulated by BMP signaling (Figures 7C,D). At stage 22HH, the *Bmp4* expression is higher in the presumptive ANZ than in the undifferentiated zone. In contrast, the *Bmp7* showed lower expression while *Bambi* did not show significant differences (Figure 7C). At stage 25HH, the expression of these genes in the ANZ was like the observed at stage 22HH (Figure 7D). We also evaluated the expression of *Msx2* and *Id2*, two genes that are regulated by BMP signaling. In the presumptive ANZ, the expression level of *Msx2* was lower than the undifferentiated zone, whereas *Id2* did not show changes in both regions. In contrast, the expression of *Msx2* was elevated in the ANZ regarding the undifferentiated zone (Figure 7C). *Id2* expression levels increased at stage 25HH in the ANZ compared with the undifferentiated zone, but at stage 22HH, no differences were found (Figure 7C). Comparing the presumptive ANZ with the ANZ, *Msx2* and *Id2* presented higher expression levels in the ANZ (Figure 7D). In contrast, the levels of *Bmp4* and *Bmp7* were lower, while Bambi is similar in both developing stages (Figure 7D).

The next group of genes evaluated is either regulated by BMP and FGF signaling or both (Figures 7E,F). *Mario* is a gene associated with the formation of digit 2, and it is induced by FGF and inhibited by BMP (Amano and Tamura, 2005). *Snail* is a transcription factor related to areas of undifferentiated mesenchyme and cell death; BMP and FGF signaling induce both genes (Ros et al., 1997; Montero et al., 2001). Results showed that the expression levels of both genes in the presumptive ANZ and ANZ are higher than in the undifferentiated zone (Figure 7E). Comparing the expression levels between the presumptive ANZ and ANZ, it was found that *Snail* expression is higher than *Mario* (Figure 7F).

## Discussion

The AER is a signaling center where FGF, WNT, and BMP signaling pathways play an essential role in controlling cell proliferation, cell survival, and cell differentiation (Fernandez-Teran and Ros, 2008; Ten Berge et al., 2008; Mariani et al., 2017). During sculpturing of the limb, mesodermal cells underneath AER in the anterior margin of the limb undergo cell death giving rise to the ANZ that appears gradually, in coordination with the progressive loss of the AER (Todt and Fallon, 1987; Fernandez-Teran et al., 2006; Fernandez-Teran and Ros, 2008).

The intricate regulatory network between BMP, FGF, and WNT signaling that controls cell death in interdigital tissue is well known (Ganan et al., 1996; Pizette and Niswander, 1999; Danopoulos et al., 2013; Haro et al., 2014; Jin et al., 2018). However, the first events triggered by this regulatory network to initiate cell death in the anterior limb undergoing ANZ formation are not well established. The present study aimed to elucidate the earliest events triggered by the regulatory network of FGF, WNT, and BMP signaling in the control of cell death to induce the ANZ formation in the anterior margin of the limb. Previously, it was reported that a short pulse of BMP is sufficient to trigger cell death in the anterior margin of the limb (Abarca-Buis et al., 2011). An interval of two to 8 h is sufficient to induce cell death after the inhibition of WNT or FGF or the activation of BMP signaling. Besides, cell death induction is coordinated with the regulation of *Dkk*, *Fgf8,* and *Bmp4* expression. Inhibition of FGF signaling inhibited *Dkk* expression after 2 h of treatment, demonstrating that FGF is necessary to induce *Dkk* gene expression. In contrast, DKK treatment-induced cell death after 8 h. Likewise, DKK and BMP4 inhibit *Fgf8* expression; thus, it is possible to postulate that the negative feedback loop between *Bmp4*, *Fgf8*, and *Dkk* controls the onset of cell death in the ANZ.

It has been demonstrated that the expression of *Fgf8* in the anterior AER is not redundant with other *Fgf* genes expressed in posterior AER (Moon and Capecchi, 2000; Moon et al., 2000; Delgado et al., 2008). Massive cell death in the anterior margin results from BMP activation (Yokouchi et al., 1996). The mutant mouse for *Bmpr1a* demonstrates that this receptor mediates BMP signaling in controlling *Fgf8* expression (Pajni-Underwood et al., 2007). Interestingly in the posterior region of the limb deprived of SHH, signaling massive cell death occurs concomitantly with up-regulation of *Bmp4* (Sanz-Ezquerro and Tickle, 2000). The absence of SHH signaling increases the repressor form of GLI3 (GLI3R), which regulates *Bmp* expression as observed in the anterior margin; GLI3R is abundant and correlates with an increase in *Bmp4* expression (Bastida et al., 2004).

In this work, comparing the expression profile of *Fgfr1* and *Fgfr2* between the presumptive ANZ with the undifferentiated region at stage 22 HH showed differential expression. *Fgfr1* expression is higher than *Fgfr2* in the presumptive ANZ. In addition, the lower levels of expression of *Churchill* or *Mkp3* that are FGF signaling targets might suggest that minimal amounts of *Fgfr1* or *Fgfr2* are enough to avoid cell death. Furthermore, FGF signaling may be active at low levels before ANZ formation. However, the levels of *Bmp4* might be the result of a regulation of GLI3R (Bastida et al., 2004), and it is possible that although higher expression of *Id2* and *Msx2* together with the low level of *Mkp3* expression observed in the anterior margin of the limb might be not sufficient to induce cell death. Thus, the balance of FGF and BMP signaling may favor FGF signaling.

As development progress, the ANZ is established. At stage 25HH, the expression levels of *Fgfr1* and *Fgfr2* and *Churchill* increase. However, the *Mkp3* level maintains slightly higher in the well-established ANZ than in the undifferentiated region. Interestingly, levels of *Churchill* are higher, which might indicate that FGF signaling is being inhibited (Kok et al., 2010). Other genes such as *Mario* and *Wif* (data not shown) are higher expressed in ANZ, BMP, whereas FGF signaling induces Mario. It has been involved in the boundary between non-digit digit two in the chick embryo (Amano and Tamura, 2005), whereas *Wif* is an inhibitor of WNT/β catenin. *Wif* together with DKK may inhibit WNT signaling and, consequently, FGF signaling, promoting cell death. The levels of expression of *Bmp7* and *Bambi* in both stages are lower than the undifferentiated zone indicating this minor participation in establishing ANZ.

Finally, comparing gene expression levels between the presumptive ANZ at stage 22HH and the ANZ of 25HH demonstrates that FGF and BMP signaling is more active in stage 25HH when the ANZ is well-established. These data suggest that the control of FGF and BMP signaling is necessary to regulate cell death.

**FIGURE 8:**
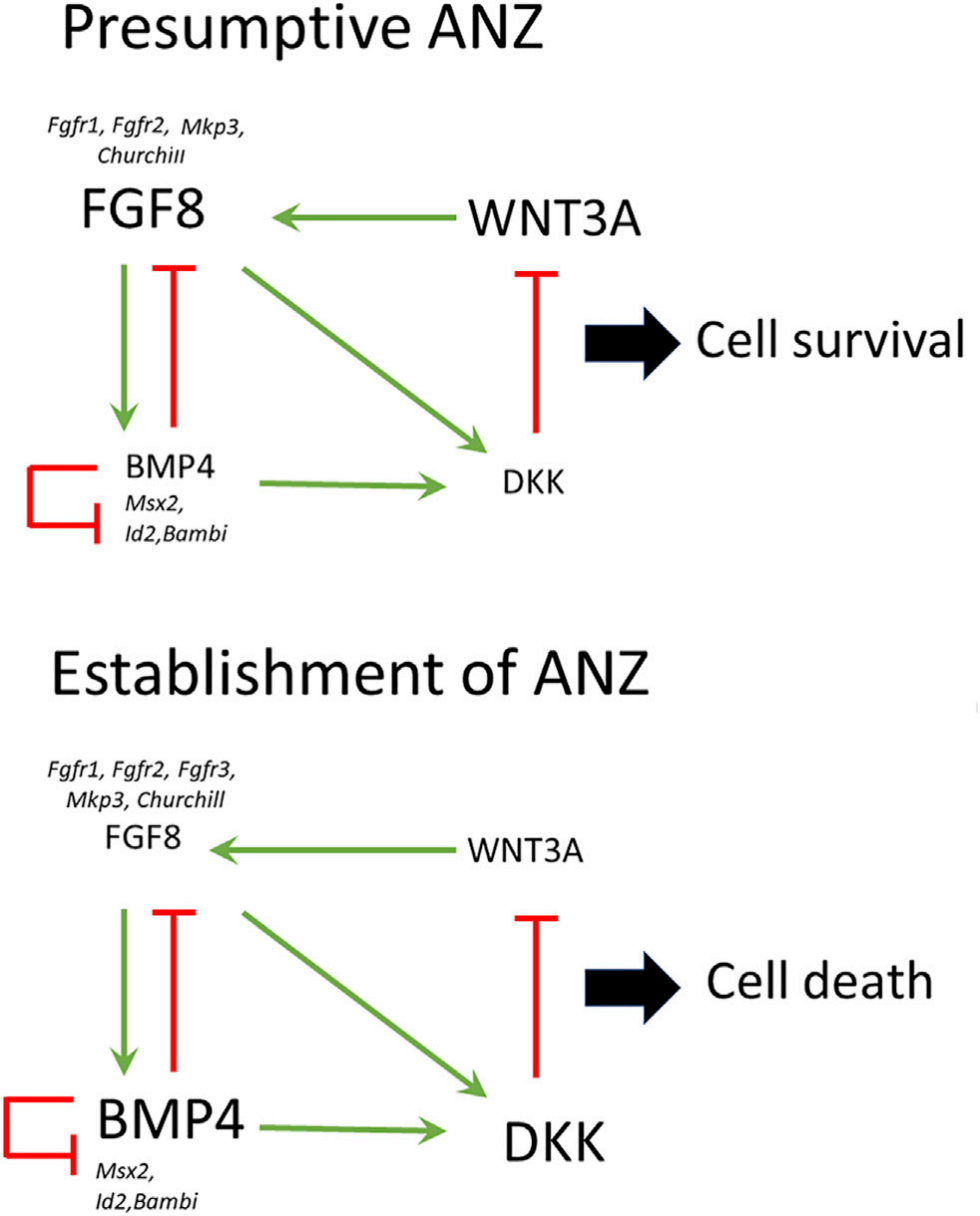
Establishment of the ANZ by the WNT-BMP-FGF regulatory network. In this model, WNT3A and BMP signaling antagonistically regulate *Fgf8* expression and consequently cell death. WNT3A induces the expression of *Fgf8* in the AER. BMP signaling inhibited *Fgf8* expression; meanwhile, DKK inhibits the function of WNT/ß catenin signaling, and consequently, the expression of *Fgf8* is inhibited. In the presumptive ANZ, the high levels of FGF8 from AER are necessary for cell survival, but FGF8 is also required for promoting cell death regulating *Bmp4* and *Dkk* expression. The establishment of ANZ occurs when the levels of FGF signaling are reduced and BMP signaling increases leading to the inhibition of *Fgf8* in the AER inducing cell death. *Fgfr1, Fgfr2, Mkp3, Churchill, Msx2, Id2, and Bambi* are expressed differentially in the presumptive ANZ and the established ANZ.

Based on data obtained in the present study and from the literature, we propose the following model to explain the onset of cell death in the anterior margin of the limb to give rise to ANZ (Figure 8). First, it is known that WNT3A mediated by *Sp6/Sp8* induces the *Fgf8* expression in the AER (Haro et al., 2014). On the other hand, BMP signaling inhibited *Fgf8* expression. Thus, WNT3A and BMP signaling antagonistically regulate *Fgf8* expression and consequently cell death (Hernandez-Martínez et al., 2009). Because *Bmp4* and other genes such as *Churchill* and *Mkp3* depend on FGF signaling (Kawakami et al., 2003), it is possible to suggest that the extent and location of cell death depend on the capacity of FGF signaling to control the levels of *Bmp4* expression. High levels of BMP signaling, presumably BMP4, inhibit *Fgf8* in a higher extension of the AER; consequently, cell death occurs. Also, BMP4 induces *Dkk* expression, and thus it is reasonable to speculate that if the levels of BMP signaling are high, then high levels of DKK are present in the anterior margin of the limb. DKK inhibits the function of WNT/ß catenin signaling resulting in an inhibition of FGF8 signaling. Remarkably as FGF signaling presumably, FGF8 from AER is necessary for cell survival but is also required for promoting cell death because it promote*s Bmp4* and *Dkk* expression. Thus, different levels of FGF activity may control the negative loop to promote AER regression and, consequently, the onset of cell death. As limb development progress, this negative feedback loop occurs progressively. Other genes such as *Msx2* and *Bambi,* although necessary for cell death (Montero et al., 2001), seem not to be regulated during the early establishment of the ANZ.

In conclusion, this work adds new insights to comprehend the establishment of a regulatory network by FGF, WNT, and BMP signaling to induce cell death in anterior mesodermal cells establishing the ANZ.

## Data Availability

The original contributions presented in the study are included in the article/Supplementary Material, further inquiries can be directed to the corresponding author.
